# Reply to Hamilton and Konvalinka: Distinguishing behavioral similarity from dyadic neural-state dynamics in hyperscanning

**DOI:** 10.1073/pnas.2626427123

**Published:** 2026-09-21

**Authors:** Peng Zhang, Xue Yang, Yi Jiang, Chunyan Guo

**Affiliations:** ^a^ Beijing Key Laboratory of Learning and Cognition, School of Psychology, Capital Normal University, Beijing 100048, China; ^b^ State Key Laboratory of Cognitive Science and Mental Health, Institute of Psychology, Chinese Academy of Sciences, Beijing 100101, China; ^c^ Department of Psychology, University of Chinese Academy of Sciences, Beijing 100049, China

We appreciate Hamilton and Konvalinka’s thoughtful discussion of behavioral similarity in driving interbrain neural synchrony (INS). Their letter highlights a widely recognized issue in hyperscanning: shared sensory, motor, and task-related processes can contribute to correlated neural activity between individuals. We agree that behavioral coordination is a meaningful contributor to INS (1–3) and that appropriate controls, such as pseudo-pairs and behavior-matched trials, are essential for interpreting synchrony measures.

However, the behavioral account proposed by Hamilton and Konvalinka addresses only one level of interpersonal neural dynamics and does not fully capture the dyadic neural-state structure characterized in our study. Their argument focuses on temporal alignment of event-related desynchronization (ERD) signals, which may influence synchrony metrics that quantify similarity between individual neural responses. In contrast, our Quantum Aligned-Misaligned Entanglement Model (QAEM) examines a different phenomenon: the relational organization of two interacting neural systems. QAEM uses phase-based coupling measures, including PLV and WPLI, to characterize how two brains jointly occupy distinct dyadic neural states rather than how similar their independent time series appear (4, 5). Thus, ERD alignment and QAEM-derived dyadic neural-state alignment represent complementary but conceptually distinct aspects of interpersonal neural dynamics.

Our experimental findings further suggest that behavioral similarity alone cannot account for the observed dyadic neural-state patterns. In Experiment 1, cooperative and independent encoding conditions produced distinct QAEM states even though both conditions involved identical sensory inputs and similar task demands. This suggests that cooperative interaction is associated with a specific organization of interbrain dynamics beyond temporal behavioral alignment (6). More importantly, Experiment 2 demonstrated that experimentally enhanced social-cognitive engagement through an empathy-based intervention altered QAEM states under an identical cooperative memory paradigm. These findings show that interpersonal factors such as social motivation and cognitive engagement can modulate dyadic neural-state configuration independently of motor timing or behavioral consistency. Consistent with the two-body neuroscience framework, social interaction involves reciprocal coupling between individuals that cannot be fully reduced to similarity between isolated neural responses (7, 8).

We agree with Hamilton and Konvalinka that pseudo-pair analyses and behavior-matched controls provide valuable approaches for disentangling shared task processing from interpersonal coupling (9, 10). Such controls are particularly important for interpreting conventional INS measures, where shared sensory inputs or task demands may generate correlated neural responses. However, QAEM is not designed to determine whether two individuals exhibit similar neural activity; rather, it aims to characterize whether interacting brains occupy distinct relational states during cooperation. Future studies integrating pseudo-pair controls, behavioral matching, and dynamic state modeling will help clarify how shared processing and interpersonal coupling jointly shape dyadic neural dynamics.

In summary, we view behavioral synchrony and dyadic neural-state alignment as complementary levels of analysis. Behavioral coordination may contribute to INS through shared processing, whereas QAEM extends this perspective by modeling the probabilistic relational states that emerge when two brains interact, providing a foundation for future computational approaches such as QAEM-Dyadic Brain Twin models (Fig. 1). Recognizing these levels of analysis will help advance hyperscanning research toward a more comprehensive understanding of interpersonal neural mechanisms.

**Fig. 1.**
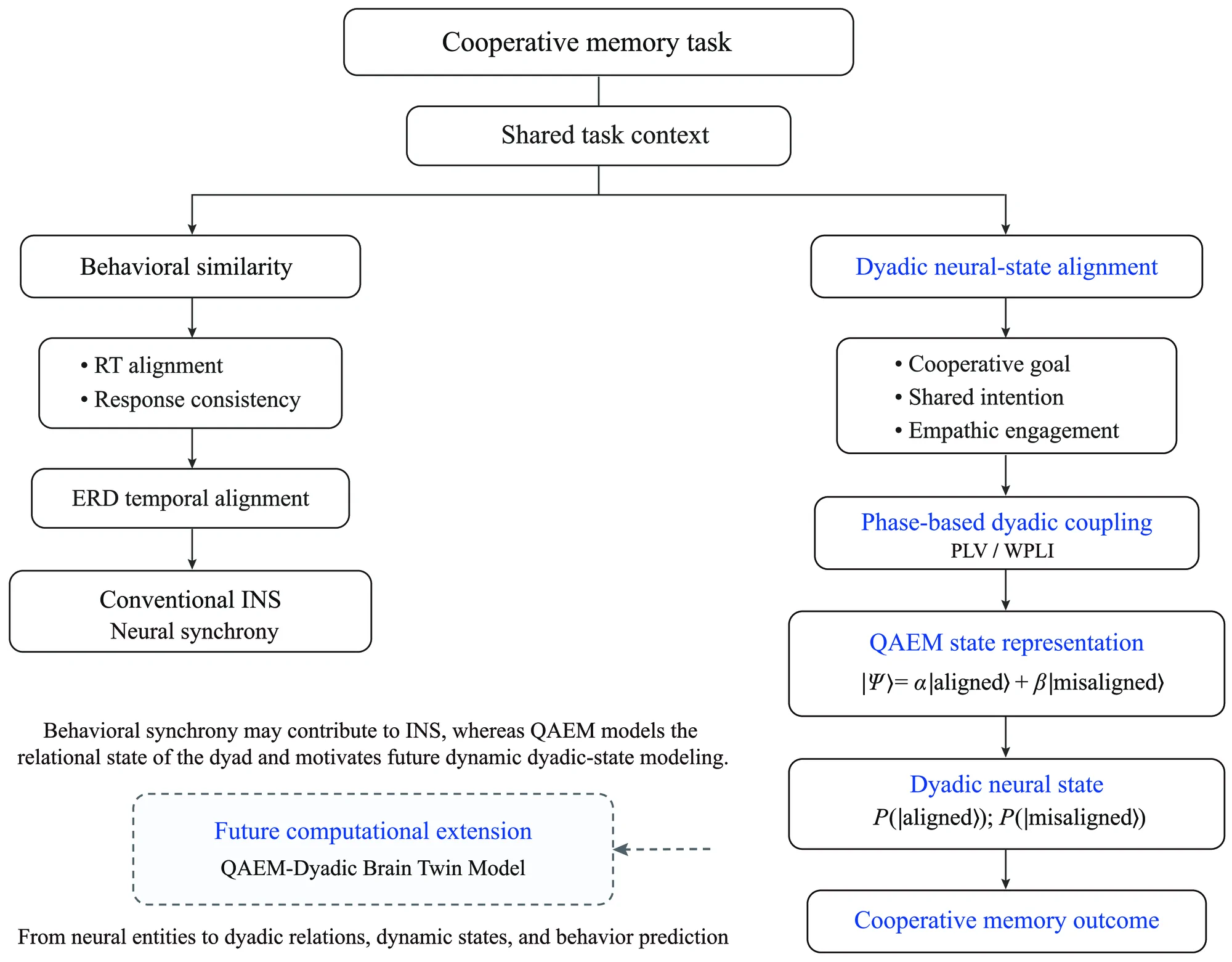
Conceptual distinction between behavioral synchrony and QAEM-based dyadic neural-state alignment.

## Data Availability

Previously published data were used for this work (https://osf.io/afq2p/) (11).
